# Intraoperative Asystole Induced by Intramedullary Reaming During Orthopedic Surgery

**DOI:** 10.7759/cureus.87739

**Published:** 2025-07-11

**Authors:** Jacob McCleerey, Gina DiMattia, Takor Arrey-Mbi

**Affiliations:** 1 Internal Medicine, Madigan Army Medical Center, Tacoma, USA; 2 Cardiology, Madigan Army Medical Center, Tacoma, USA

**Keywords:** asystole, diaphyseal fracture, hip surgery, intramedullary nail, intramedullary reaming

## Abstract

Intramedullary nailing has become the standard of care for the treatment of femoral diaphyseal fractures due to its low incidence of complications and return of limb function. We present a case of a rare complication that we believe deserves attention for further studies and research. A 71-year-old female with a past medical history of essential hypertension, hypothyroidism, and osteoarthritis of the bilateral knees presented after a ground-level fall resulting in a right intertrochanteric femoral fracture. The patient underwent operative repair with an intramedullary nail during orthopedic surgery. Preprocedural vitals were stable, and no abnormalities were seen on preprocedural EKG. During the procedure, the patient experienced two episodes of asystole that began with intramedullary reaming and spontaneously resolved with cessation of reaming. The patient remained hemodynamically stable throughout the procedure, and as the patient’s asystole acutely resolved to normal sinus rhythm after intramedullary reaming was stopped, no acute interventions were necessary, such as cardiopulmonary resuscitation or pharmacological interventions. The procedure was successfully completed. The patient had an unremarkable postoperative course and was discharged on postoperative day one. The suspected etiology of this finding is a parasympathetic-mediated bradycardia caused by transmitted pressures from the reaming through the hip joint and into the abdominal cavity. Only a few case reports have focused on intraoperative asystole associated with intramedullary reaming. In this case report, we aim to present another case of this rare occurrence and discuss current hypotheses of the physiology, with the main objective of recognizing transient asystole as a possible complication of intramedullary reaming and suggesting further research into understanding this physiology.

## Introduction

Intramedullary nailing is the standard of care for femoral diaphyseal fractures [1,2]. High fracture union rates, an excellent return of limb function, and a low incidence of complications make intermedullary nailing one of the most successful orthopedic procedures [1]. However, as with any surgical procedure, risks and complications exist. Some clinical complications that have been associated with intramedullary nailing include fat embolism, acute respiratory distress syndrome, infection, and dysrhythmias [1,2]. Two prior case reports of asystole during reaming of the intermedullary femoral canal have been described by Martinez et al. and Wooster et al. [3,4]. In each of these cases, the patient was noted to have severe bradycardia/asystole that resolved spontaneously after reaming stopped. We present a case of transient intraoperative asystole also triggered by right femoral intramedullary reaming with spontaneous resolution when intermedullary reaming was terminated. The objective of this case report is to describe a rare complication that has been associated with reaming during intramedullary nail procedures to bring focus to this complication for further research that will hopefully benefit both surgical procedures and patient care.

## Case presentation

A 71-year-old female with a past medical history of essential hypertension, hypothyroidism, and osteoarthritis of the bilateral knees presented to the emergency room after a ground-level fall resulting in severe right hip pain. The patient had been independently ambulatory in the past with no other history of falls, osteoporosis, or previous fractures. The patient had no prior cardiac history before her procedure and had a revised Cardiac Risk Index score of less than 1, which did not warrant any further cardiac preoperative risk assessment. Preliminary EKG showed normal sinus rhythm with no conduction abnormality present. Laboratory results were unremarkable (Table 1).

**Table 1:** Preoperative and postoperative laboratory values with reference ranges. WBC: white blood cell count; MCV: mean corpuscular volume; GFR: glomerular filtration rate; TSH: thyroid-stimulating hormone

Laboratory parameter	Pre-procedure	Post-procedure	Reference range
WBC	5.0 × 10^3^/µL	8.6 × 10^3^/µL	4.5–13.0 × 10^3^/uL
Hemoglobin	13.9 g/dL	12.7 g/dL	10.0–15.0 g/dL
Hematocrit	43%	38%	35%–45%
Platelets	289 × 10^3^/µL	250 × 10^3^/µL	140–420 × 10^3^/µL
MCV	85 fL	85 fL	80–98 fL
Sodium	140 mmol/L	138 mmol/L	135–145 mmol/L
Potassium	4.30 mmol/L	3.70 mmol/L	3.50–5.10 mmol/L
Chloride	102 mmol/L	102 mmol/L	98–107 mmol/L
Bicarbonate	27 mmol/L	22 mmol/L	22–31 mmol/L
Serum creatinine	0.63 mg/dL	0.72 mg/dL	0.5–1.00 mg/dL
Estimated GFR	95 mL/min/1.73m^2^	89 mL/min/1.73m^2^	-
Glucose	87 mg/dL	97 mg/dL	74–109 mg/dL
Calcium	9.5 mg/dL	8.9 mg/dL	8.6–10.3 mg/dL
Albumin	4.5 g/dL	4.2 g/dL	3.5–5.2 g/dL
TSH	2.27 µlU/mL	-	0.27–4.32 µlU/mL
T4	1.27 ng/dL	-	0.58–1.64 ng/dL

**Figure 1:**
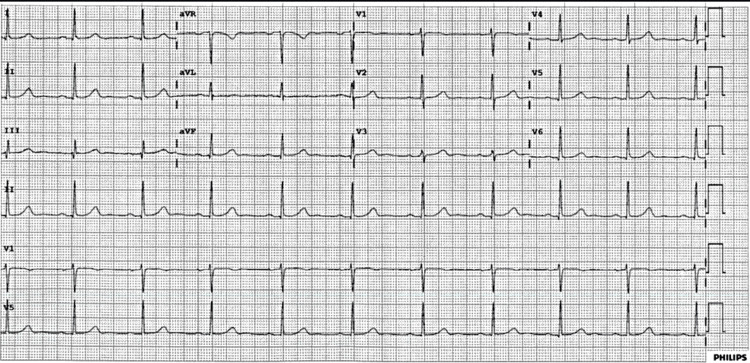
Baseline EKG showing normal sinus rhythm.

**Figure 2:**
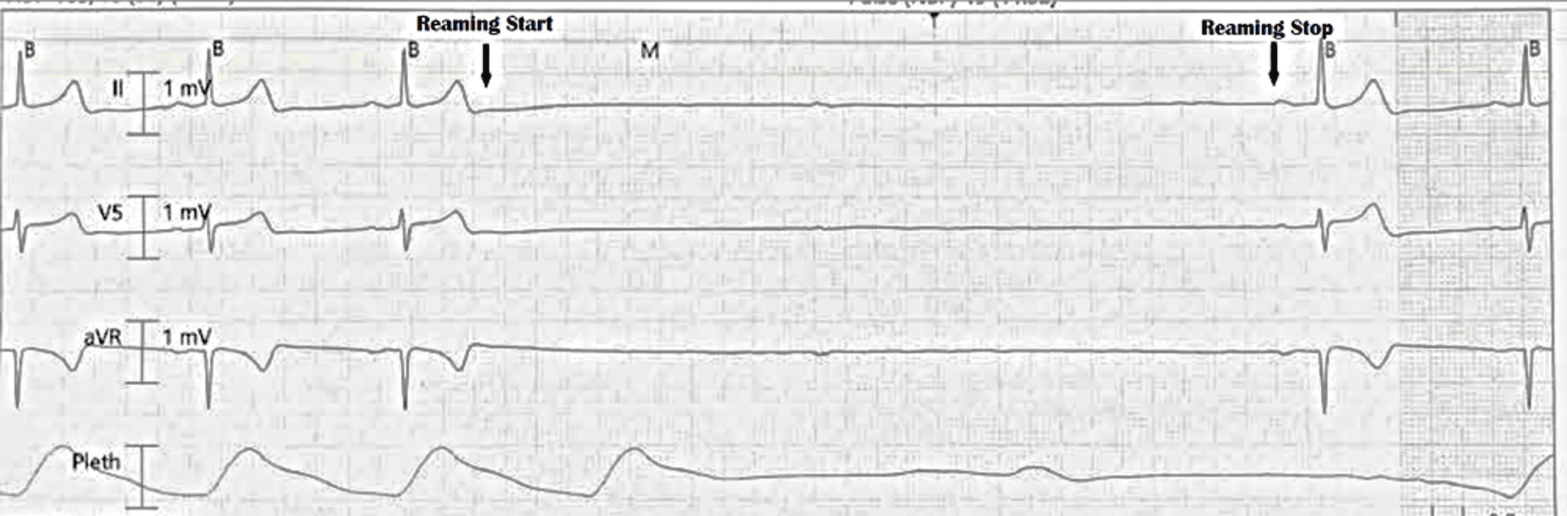
Intraoperative EKG monitoring showing reamer on and off.

She was found to have a right intertrochanteric femoral fracture visualized on X-ray and CT, which necessitated evaluation by orthopedic surgery. Orthopedic surgery recommended operative repair with an intramedullary nail. Her vitals before the start of the procedure were a heart rate of 71 beats per minute, systolic blood pressure of 152 mmHg, diastolic blood pressure of 62 mmHg, and an oxygen saturation of 97% breathing ambient air. Baseline EKG showed normal sinus rhythm (Figure 1). She remained hemodynamically stable after induction of anesthesia. However, during intramedullary reaming, the patient abruptly experienced episodes of asystole. The longest asystole episode lasted six seconds (Figure 2). The asystole episode spontaneously resolved with cessation of reaming (Figure 2).

The patient remained hemodynamically stable for the remainder of the procedure. Cardiology was consulted intraoperatively, and as the patient’s asystole acutely resolved to normal sinus rhythm after intramedullary reaming was stopped, no acute interventions were necessary, such as cardiopulmonary resuscitation or pharmacological interventions. Other etiologies for asystole, including anesthetics, acute ischemia, and fat embolism, were discussed but ruled out as the patient remained hemodynamically stable and asystole resolved upon cessation of intramedullary reaming. Limited intraoperative echocardiogram showed preserved ejection fraction with no significant left ventricular wall motion abnormality. Given the return to baseline and ruling out other possible considerations, it was decided by the surgical and cardiology teams that the procedure should continue. Cardiology was consulted postoperatively and recommended further evaluation with a full transthoracic echocardiogram (TTE). TTE demonstrated normal left ventricular size and function with an ejection fraction of greater than 65%, normal wall motion, and no evidence of valvular heart disease. Cardiac biomarkers drawn during the procedure returned normal, and the postoperative EKG was unchanged when compared to her pre-procedural EKG. She was monitored on telemetry overnight without recurrence of dysrhythmia or other conduction abnormalities and was discharged on postoperative day one.

## Discussion

Possible etiologies for intraoperative cardiac arrest can include fat embolism, acute coronary syndrome, medication effects, or others. In this case, myocardial infarction was considered but was found to be unlikely given no acute ischemic changes seen on the telemetry monitors or post-procedure EKG and normal troponin level. Additionally, fat embolism syndrome is a known potential complication of long bone fractures or surgical repair. This patient did not demonstrate traditional symptoms associated with fat embolism, such as respiratory distress, neurological symptoms after the procedure, or petechial rash. Given the patient’s hemodynamic and respiratory stability outside of the reaming period as well as the absence of post-procedure symptoms and typical clinical findings of known complications, it was suspected that this patient’s asystole was similar to the same mechanism described in the other case reports by Martinez et al. and Wooster et al. [3,4]. Intraoperative asystole during intramedullary reaming is a rare occurrence that has been scarcely reported in the literature. The proposed mechanism has been described as likely secondary to a vagal response or the Bezold-Jarisch reflex, resulting in bradyarrhythmia or asystole [2,4]. The pathophysiology of this reflex was described by Wilkinson and Gallagher [2] as transmitted pressures through the pelvis to the intra-abdominal organs, resulting in a vagal-mediated bradycardia. However, the pathophysiology of this reflex is not well documented, and we hope that by demonstrating this case report, as well as the previous reports mentioned, other mechanisms can be identified and investigated further.

## Conclusions

Multiple etiologies of the patient’s transient asystole were considered to include intraoperative cardiac event and fat embolism. However, as the patient did not have a significant previous cardiovascular history and post-procedural workup for cardiovascular complications with troponin, EKG, and TTE was normal, these etiologies were considered to be less likely. Therefore, we conclude that this case demonstrates a transient intraoperative asystole induced by intramedullary reaming, as described by earlier case reports and the Bezold-Jarisch reflex. We believe that this finding underscores the potential cardiovascular risks associated with this common orthopedic procedure and recommend that further studies be conducted to better document and evaluate this complication. Until this reflex is better understood and described as a clear cause of intraoperative asystole during reaming, we recommend that surgeons, anesthesiologists, and cardiologists be aware of this condition and prepare for this potential complication intraoperatively.
